# *Orientia tsutsugamushi* binds to multiple C-type lectin receptors

**DOI:** 10.1128/iai.00497-25

**Published:** 2026-02-09

**Authors:** Véronique Hefter, Sabine Mayer-Lambertz, Zacharias Orfanos, Jonas Mehl, Bernd Lepenies, Christian Keller

**Affiliations:** 1 Institute of Virology, Philipps University Marburg 9377, Marburg, Germany; 2 German Centre for Infection Research at the Institute of Virology, Philipps University 9377, Marburg, Germany; 3 Institute for Immunology and Research Center for Emerging Infections and Zoonoses, University of Veterinary Medicine Hannover 26556 , Hanover, Germany; 4 Institute for Biochemistry and Chemistry, Veterinary Faculty, Ludwig-Maximilians University München 9183, Munich, Germany; 5 Institute of Medical Microbiology and Hygiene, University Hospital Regensburg 39070, Regensburg, Germany; Stanford University School of Medicine, Stanford, California, USA

**Keywords:** innate immunity, C-type lectins receptors, Mincle, scrub typhus, *Orientia tsutsugamushi*

## Abstract

*Orientia tsutsugamushi*, the agent of scrub typhus, is an obligate intracellular bacterium whose atypical cell wall lacks many classical pathogen-associated molecular patterns but is enriched in neutral glycans. Its recognition by phagocytes is driven by a heat-stable ligand that triggers innate cytokine responses, yet the nature of this ligand and the receptors sensing it remain incompletely understood. While activation of innate immunity via toll-like (TLR) and nucleotide-binding and oligomerization domain-like receptors has been described, recognition via C-type lectin receptors (CLRs) has remained largely unexplored. Using a flow cytometry-based screening assay with a library of CLR-Fc fusion proteins, we demonstrate binding of *Orientia* to four mouse CLRs, including Mincle, Dectin-1, Langerin, and DCL-1, as well as to human DC-SIGN. Binding to Mincle was Ca²^+^-dependent, indicating carbohydrate-specific recognition. In bone marrow-derived dendritic cells (BMDC), heat-inactivated *Orientia* induced transcriptional upregulation of Mincle in a MyD88-dependent manner, suggesting cross talk between TLR and CLR pathways. Mincle was dispensable for TNF-α induction in BMDC stimulated with *Orientia* but contributed to the induction of *interleukin-27* and *cxcl-10* mRNA, indicating an immunomodulatory rather than a classical pro-inflammatory role. We also discuss *in vivo* data that demonstrated upregulation of Mincle and concomitant downregulation of Dectin-1 during *Orientia* infection. Together, this study identifies multiple CLRs as receptors for *Orientia*, highlights Mincle as a modulator of innate immunity, and suggests that Mincle-driven immunoregulation helps to shape the inflammatory environment during scrub typhus.

## INTRODUCTION

*Orientia tsutsugamushi* is an obligate gram-negative intracellular bacterium and the causative agent of scrub typhus, a neglected febrile disease endemic in Southeast Asia. While typically dominated by a T helper 1 (Th1)-skewed immune response (1), severe cases can be accompanied by elevated systemic concentrations of innate cytokines and chemokines, especially tumor necrosis factor (TNF)-α (2, 3).

The roles of host pattern recognition receptors (PRRs) in innate immune activation by *O. tsutsugamushi* remain, however, ill-defined. It was observed that *Orientia* provides a heat-stable ligand to induce cytokine and chemokine responses via NF-κB signaling pathways in murine macrophages (4). The absence of lipopolysaccharide (LPS) from the outer membrane, the absence of respective synthesis pathways in the bacterial genome, and direct experimental evidence suggested that the LPS-specific Toll-like receptor (TLR) 4 is not involved in this recognition (5–9). Previous work suggested an involvement of nucleotide-binding and oligomerization domain-1 (10) and TLR 2 (7).

It was found by biochemical methods that *Orientia* has an unusually high amount of neutral sugar in its outer membrane compared to other gram-negative bacteria (9). In the present study, we therefore investigated the role of C-type lectin receptors (CLRs) in recognition of *Orientia* and modulation of innate responses triggered by heat-stable *Orientia* ligands.

Myeloid CLRs are transmembrane lectins that act as PRR in innate immunity (11). They are mainly expressed by myeloid cells, such as dendritic cells (DCs), macrophages, and neutrophils, and contribute to pathogen recognition, antigen presentation, and shaping of the adaptive immune response (12, 13). Often, CLRs recognize their cognate ligands in a Ca^2+^-dependent manner; however, some CLRs lack classical Ca^2+^ binding sites, thus bind to ligands in a Ca^2+^-independent fashion (14). While the majority of CLRs were shown to bind to carbohydrate structures, including fucose and mannose (DC-SIGN, Langerin, and Dectin-2), β-1,3-glucan (Dectin-1), and trehalose derivatives (Mincle) (15), some CLRs recognize non-carbohydrate ligands, such as proteins, lipids, and even crystalline structures (16, 17). Consequently, CLRs are rather promiscuous sensors for structures from a wide range of pathogens but also endogenous ligands released by cell damage or death (18).

The CLR macrophage-inducible lectin (Mincle) was shown to be a receptor for glycolipids or glycoproteins, such as the cord factor from *Mycobacterium tuberculosis*, trehalose-6,6′-dimycolate (15, 19). Moreover, Mincle recognizes a variety of pathogens including bacteria (group A *Streptococcus* and *Legionella pneumophila* [20, 21]), fungi (*Pneumocystis carinii* [*jiroveci*] and *Malassezia* [22, 23]), parasites (*Leishmania* spp. [24]), and also endogenous ligands (25). The CLRs Dectin-1 and Dectin-2 are involved in the sensing (and control) of mycobacteria and pathogenic fungi (26–29), whereas Langerin, a receptor recognizing mannose-containing glycan structures, contributes to HIV and *Mycobacterium leprae* recognition (30, 31).

Besides uptake and internalization of microorganisms, CLR ligation by microbial ligands can induce intracellular signaling such as NF-κB-dependent gene transcription. This activating pathway involves binding of the intracellular Syk kinase either to hemITAM motifs in the CLR cytoplasmic tails as shown for Dectin-1 or to cytoplasmic ITAMs within the associated FcRγ chains (Mincle and Dectin-2) (13). Alternatively, immune-dampening functions have been associated with CLR signaling, such as signaling via inhibitory ITAMs and binding of the SHP-1 phosphatase (24). In addition, cross-talk mechanisms between CLRs and other PRRs, such as TLRs, can augment or modulate CLR-mediated signaling, as shown for instance for DC-SIGN and TLR4 (32, 33).

The interaction between CLRs and *Orientia* is not well characterized. Recent observations from experimental mouse infections suggest that CLRs could be involved in the host response toward *Orientia*: Mincle was among the most upregulated genes in the lungs of mice infected with the Karp strain (10 days post-infection [p.i.]). Other CLR genes that were upregulated in lung tissue during lethal *Orientia* infection included Clec4d (MCL), Clec5a (MDL-1), and Clec12a (MICL), whereas Clec4b1 (DCAR2), Clec7a (Dectin-1), and Clec9a (DNGR-1) were downregulated (summary in Table S1) (34). The role of Mincle was studied further by Fisher et al. (34), but since only limited colocalization between Mincle and intracellular *Orientia* antigens was observed, it has remained unknown whether and which CLRs, including Mincle, directly interact with *Orientia*.

In the present study, we used a comprehensive library of CLR-Fc fusion proteins (35, 36) and established a flow cytometric approach to investigate the direct interaction of *Orientia* with six CLRs from mice and one human CLR. Moreover, we demonstrate TLR-mediated signals as a requirement for Mincle induction by heat-stable *Orientia* and show that Mincle modulates the transcriptional program of several cytokines involved in *Orientia*-induced inflammation.

## MATERIALS AND METHODS

### Isolation and cultivation of BMDC

C57BL/6 wild-type mice were kindly provided by Stefan Bauer, Institute of Immunology, Philipps University Marburg. *MyD88*^−/−^ and C57BL/6 wild-type control animals were kindly provided by Carsten Kirschning, Institute of Medical Microbiology, University Hospital Essen. The *Mincle*^−/−^ mouse line was obtained from the National Institutes of Health-sponsored Mutant Mouse Regional Resource Center National System and was back-crossed with C57BL/6 mice for >10 generations. *Mincle^−/−^* mice and C57BL/6 wild-type control mice were kept at the animal facility at the University of Veterinary Medicine Hannover, Germany, under controlled and specific pathogen-free conditions. Preparation of bone marrow-derived dendritic cells (BMDC) was performed as previously described (7). Briefly, bone marrow cells were flushed from the femur and tibia with warm medium and differentiated in RPMI medium, in the presence of 10 ng/mL recombinant mouse GM-CSF (Peprotech, Cranbury, NJ, USA) or 10% supernatant from X63-Ag 8653 myeloma cells that are stably transfected with a plasmid coding for GM-CSF. Cells received new medium on days 3 and 6 and were collected for experiments on day 7.

### Cell culture

HEK-Blue mMincle cells (InvivoGen, San Diego, CA, USA) were maintained in Dulbecco’s Modified Eagle Medium supplemented by selective antibiotics, as per the manufacturer’s instructions. For experimental purposes, HEK-Blue mMincle cells were resuspended in HEK-Blue Detection (InvivoGen) and seeded at 0.05 × 10^6^ cells/well in 96-well plates. Reading of optical density was performed on a spectrophotometer (Emax Precision microplate reader, Molecular Devices, San Jose, CA, USA) at 650 nm at indicated time points.

### Bacterial culture

*O. tsutsugamushi* Karp strain was kindly provided by J. Stenos (Australian Rickettsial Reference Laboratory, Geelong, Australia). Bacteria were cultured under BSL3 conditions at Philipps University Marburg in γ-irradiated L929 cells (DSMZ, Braunschweig, Germany; 10 Gy delivered by X-rays using an X-RAD 320 ix instrument [Precision X-Ray Inc., North Branford, CT, USA]) for 7 days before subculturing. RPMI medium was supplemented with 5% fetal calf serum (FCS) and 4 mM L-glutamine. All experiments were performed using bacteria harvested from continuous culture 7 days p.i.

To generate a purified bacterial suspension for experimental procedures, infected cells were disrupted by vortexing for 6–10 min using sterilized glass beads (diameter 3 mm, VWR, Darmstadt, Germany). The suspension was then filtered using a 2 µm filter (Whatman, Maidstone, UK) and centrifuged at full speed for 20 min to pellet cell-free bacteria. The pellet was resuspended in cell culture media for further experiments. All inactivation procedures were performed on a neoBlock Heater Mono I instrument (NeoLab, Heidelberg, Germany) by heating the suspension at 70°C for 30 min if not stated otherwise.

### BMDC stimulation assay

BMDC were seeded in 0.2 mL medium at 0.05 × 10^6^ cells/well in 96-well plates (enzyme-linked immunosorbent assay [ELISA] experiments), or in 1 mL medium at 0.5 × 10^6^ cells/well in 12-well plates (RNA experiments). LPS (*Salmonella enterica* Minnesota Re595, Sigma-Aldrich, Hamburg, Germany) and trehalose-6,6-dibehenate (TDB; Invivogen, Toulouse, France) were added in medium to reach the indicated final concentrations. Neutralization of Mincle was performed using a monoclonal rat-anti-Mincle antibody (clone 6G5, IgG2b, Invivogen, San Diego, USA) at a concentration of 1.0 µg/mL for 1 h. Rat IgG (1.0 µg/mL; Sigma-Aldrich, Hamburg, Germany) was used as control.

### Enzyme-linked immunosorbent assay

TNF-α concentration from cell culture supernatants was measured by sandwich ELISA using ELISA MAX Deluxe Kits (BioLegend, San Diego, USA), according to the manufacturer’s instructions. Reading was performed on a spectrophotometer (Emax Precision microplate reader (Molecular Devices, San Jose, CA, USA) at 450 nm (reference channel 650 nm).

### mRNA transcription analysis by quantitative reverse transcription polymerase chain reaction (qRT-PCR).

Total RNA from cell culture samples was extracted using RNeasy Kit (Qiagen, Hilden, Germany), following the manufacturer’s instructions, followed by an on-column DNase digestion with RNase-free DNase Set (Qiagen, Hilden, Germany). Reverse transcription to complementary DNA (cDNA) was performed with a high-capacity cDNA reverse transcription kit (Applied Biosystems, ThermoFisher Scientific, Karlsruhe, Germany). cDNA was stored at −20°C until use. Quantification of mRNA transcription was performed using HotStarTaq polymerase (Qiagen, Hilden, Germany) on a LightCycler 480 II instrument (Roche, Mannheim, Germany). In a total volume of 10 µL, the PCR mastermix contained 200 µM deoxynucleotide triphosphates, 2.5 mM MgCl_2_, 0.3 µM sense and antisense primers (see Table 1 for sequences), 0.1 µL of a 1:1,000 dilution of SYBR Green (Invitrogen, Thermo Fisher Scientific, Darmstadt, Germany) in dimethyl sulfoxide, 0.25 U Taq DNA Polymerase, and 1 µL template cDNA. Reactions were run in duplicates. Enzyme activation at 95°C for 15 min was followed by 39 cycles of 30 s at 95°C, 40 s at 58°C, and 30 s at 72°C, with touchdown from 64°C to 58°C in cycles 1 to 6. Gene expression was normalized to the housekeeping gene RPS9 using the 2^−ΔΔCt^ method.

**TABLE 1:** Oligonucleotide sequences used in relative gene expression analysis

Primer target	Primer sequence	Reference
Ccl-2	FW 5′–TCTCTCTTCCTCCACCACCA–3′	(37)
	RV 5′–CGTTAACTGCATCTGGCTGA–3′	
TNF-α	FW 5′–GTTTGCTACGACGTGGGCT–3′	(37), modified
	RV 5′–CCAAATGGCCTCCCTCTCA–3′	
Cxcl-9	FW 5′–GGAGTTCGAGGAACCCTAGTG–3′	(38)
	RV 5′–GGGATTTGTAGTGGATCGTGC–3′	
Cxcl-10	FW 5′–AGTGCTGCCGTCATTTTCTG–3′	(39)
	RV 5′–ATTCTCACTGGCCCGTCAT–3′	
IL-27 p28	FW 5′–CTGTTGCTGCTACCCTTGCTT–3′	(40)
	RV 5′–CACTCCTGGCAATCGAGATTC–3′	
Mincle	FW 5′–TGCTACAGTGAGGCATCAGG–3′	(41)
	RV 5′–GGTTTTGTGCGAAAAAGGAA–3′	
RPS9	FW 5′–CCGCCTTGTCTCTCTTTGTC–3′	(42)
	RV 5′–CCGGAGTCCATACTCTCCAA–3′	

### Generation of the CLR-hFc fusion protein library

The production of the CLR-hFc fusion proteins was performed as described by Maglinao et al. (36). In short, RNA isolated from the spleen of mice was reverse transcribed into cDNA using a reverse transcriptase (New England Biolabs, Ipswich, USA). To amplify the cDNA encoding the extracellular part of each CLR, a PCR was performed using specific primers (see Table 2). The obtained cDNA fragments were ligated into a pFuse-hIgG1-Fc expression vector (InvivoGen, San Diego, USA), and CHO-S cells were subsequently transiently transfected with these vector constructs using MAX reagent (InvivoGen). After 4 days of transfection, the cell supernatant harboring the CLR-hFC fusion proteins was collected, and fusion proteins were purified using HiTrap Protein G HP columns (GE Healthcare, Piscataway, USA). The obtained proteins were analyzed by sodium dodecyl sulfate polyacrylamide gel electrophoresis and subsequent Coomassie staining as well as Western blot using an anti-human IgG-horseradish peroxidase antibody (Dianova, Hamburg, Germany).

**TABLE 2:** Oligonucleotide sequences used for cloning of recombinant CLRs

CLR	Cloning primer
mMincle	FW 5′–CCATGGGGCAGAACTTACAGCCACAT–3′
	RV 5′–AGATCTGTCCAGAGGACTTATTTCTG–3′
mDectin-1	FW 5′–GAATTCTTCAGGGAGAAATCCAGAGG–3′
	RV 5′–AGATCTTGAAGAAGTATTGCAGATTTGGTT–3′
mDectin-2	FW 5′–CCATGGAGAAAACATCATTCCAGCCCC–3′
	RV 5′–GAATTCCTGGAGCACCAGTGAGCAGAAC–3′
mCLEC9a	FW 5′–GAATTCGGGCATCAAGTTCTTCCAGGTATCC–3′
	RV 5′–CCATGGTGCAGGATCCAAATGCCTTCTTC–3′
mLangerin	FW 5′–GATATCAGGTCGTGTGGACGATGCTGAGGT–3′
	RV 5′–CCATGGTTTGGACGTAGGGCCTCTTGCAG–3′
mDCL-1	FW 5′–GAATTCCTGTCCTTCATCTACCTGGGTCC–3′
	RV 5′–CCATGGTGTCATATGGGATTGCTGCTTT–3′
hDC-SIGN	FW 5′–GAATTCGTCCAAGGTCCCCAGCTCCAT–3′
	RV 5′–CCATGGACGCAGGAGGGGGGTTTGGGGT–3′

### Binding studies: flow cytometry

Binding of CLR-hFc fusion proteins to *O. tsutsugamushi* Karp was analyzed using heat-inactivated bacteria. Bacteria were incubated with a 2F2 monoclonal antibody (mouse anti-56kDa, 1:200) (43) at 37°C for 30 min, followed by detection with a goat anti-mouse-Fc-AlexaFluor (AF)488 antibody (1:200, Abcam, Cambridge, England). Binding to CLR-hFc fusion proteins was performed as previously described (35). Briefly, phosphate-buffered saline-washed samples were incubated for 1 h at 4°C with 200 ng of the respective fusion protein in lectin binding buffer (50 mM HEPES, 5 mM MgCl_2_, and 5 mM CaCl_2_) or buffer containing EDTA (10 mM EDTA and 50 mM HEPES). Samples were washed two times with lectin-binding buffer or buffer containing EDTA and incubated for 25 min at 4°C with 1:300 diluted goat anti-human-Fc-AF680 antibody (Dianova, Hamburg, Germany). Flow cytometric measurements were performed on an Attune NxT Flow Cytometer (Thermo Fisher Scientific, Darmstadt, Germany). hFc protein was used to exclude non-specific binding of the bacteria to the Fc part of the CLR-Fc fusion proteins.

### Statistical analysis

Data were analyzed using the GraphPad Prism v9.0.1 and FlowJo v10.7.1 software. A *P*-value of <0.05 was considered significant.

## RESULTS

### Interaction of *Orientia* with recombinant mouse and human lectin-hFc

**Fig 1:**
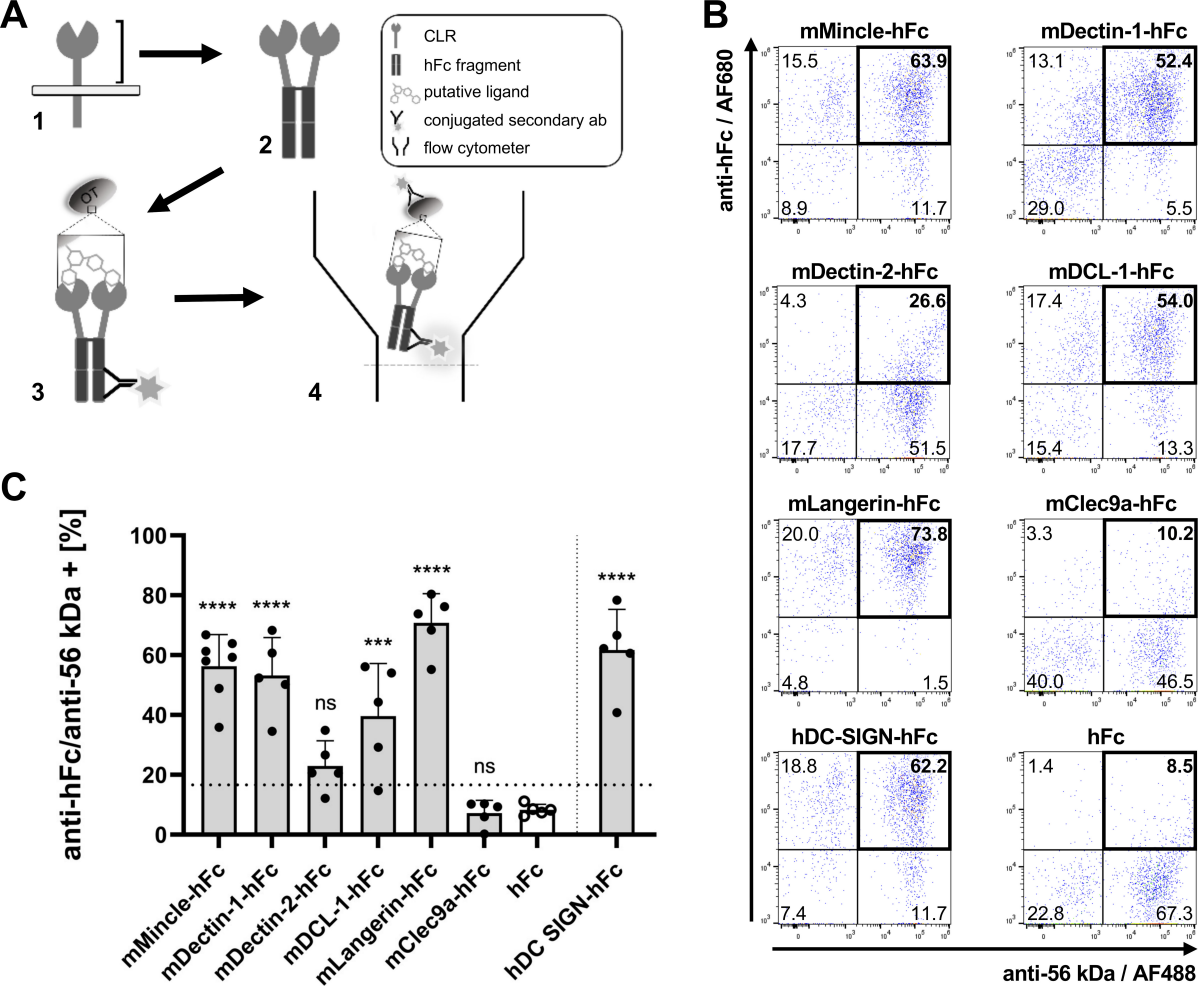
Human and mouse CLR-hFc bind to *O. tsutsugamushi*. (**A**) Construction of CLR-hFc fusion protein library. (1) Cloning the extracellular domains of CLRs. (2) fusion of hFc. (3) Binding of CLR-hFc fusion proteins to glycans exposed on bacteria, detected by anti-hFc antibodies. (4) Detection of binding by flow cytometry. (**B, C**) 70°C-inactivated purified *Orientia* were incubated with six mouse (m) and one human (h) CLR-hFc and co-stained with an *Orientia*-specific mouse anti-56 kD mAb, goat anti-mouse-Fc-AF488 Ab, and goat anti-human IgG-Fc-AF680 Ab. Binding was measured by flow cytometry. (**B**) Representative dot plots from one out of five independent experiments. (**C**) Pooled data from five independent experiments (mMincle: two experiments with two replicates, three experiments with one replicate; all others: one replicate per experiment). Means ± SD, ****, *P* < 0.0001; ***, *P* < 0.001; ns, no significant difference by one-way analysis of variance followed by Dunnett’s multi-comparison test, comparing CLR-Fc fusion protein binding to the hFc control. The twofold mean of the hFc control is depicted as a cutoff value (dotted line).

With respect to the reported pronounced heat stability of the innate ligand of *Orientia* (44), we hypothesized that the heat-stable ligand could be of carbohydrate nature. To identify interactions between inactivated, cell-free *Orientia* organisms and mammalian CLRs, we used a library of six recombinant mouse and one human CLR-hFc fusion constructs (Fig. 1A). Binding of CLRs to the surface of *Orientia* was investigated by flow cytometry (Fig. 1A) after double-staining using a mAb directed against the 56 kD antigen of *Orientia* (43). Bacteria staining double-positive for both anti-56 kD and anti-hFc (indicating binding of the respective CLR-hFc fusion protein) were considered as binding (Fig. 1B). While background staining accounted for approximately 10% of events, mean percentages of >50% positive events were found for the three CLR-hFc-fusion proteins mMincle, mDectin-1, and mLangerin, the stainings for mMincle and mLangerin being strongest (Fig. 1C). Although at a somewhat lower level (20%–60%), significant binding was also found for mDCL-1. Binding of *Orientia* to mDectin-2 was more variable (15%–40%) and not significantly higher compared to the hFc control, and no interaction was detected for mClec9a (Fig. 1C). With a mean of 60% double-positive events, the human CLR DC-SIGN also interacted with *Orientia*. Thus, we identified strong interaction of four mouse and one human CLR-hFc fusion protein with purified *Orientia*.

### Binding of Mincle to *Orientia* requires Ca^2+^

**Fig 2:**
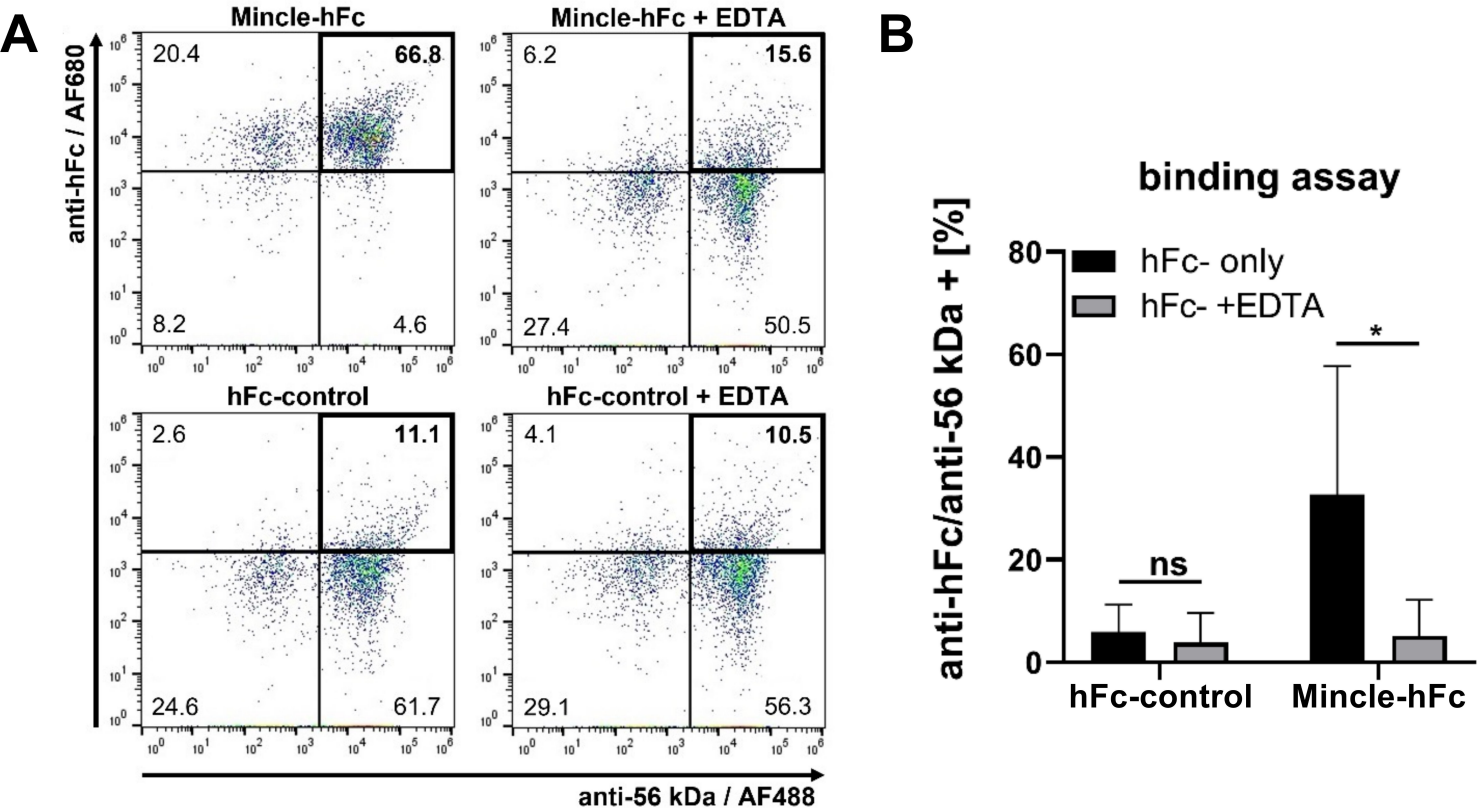
EDTA abrogates binding of *O. tsutsugamushi* to Mincle-hFc. (**A**) 70°C-inactivated purified *Orientia* were incubated with mMincle-hFc or hFc-control with (right) and without (left) EDTA buffer, and binding was measured by flow cytometry. Dot plots show representative data from one out of three independent experiments. (**B**) Means ± SD from pooled data of three independent experiments. One technical replicate per experiment was measured; for Mincle-hFc, one of three experiments was performed in technical duplicate. *, *P* < 0.05; ns, no significant difference (by two-way analysis of variance).

We selected the Mincle receptor, which had shown to be among the most strongly upregulated genes in experimental *Orientia* infection (34), for further *in vitro* characterization of the interaction with *Orientia*. First, we demonstrated that in the presence of the chelating agent EDTA, binding of Mincle to *Orientia* was reduced to background levels (Fig. 2A and B), suggesting that Mincle binds to *Orientia* at its Ca^2+^-dependent primary sugar binding site.

### Induction of *mincle* mRNA transcription in BMDC by *Orientia* depends on MyD88

**Fig 3:**
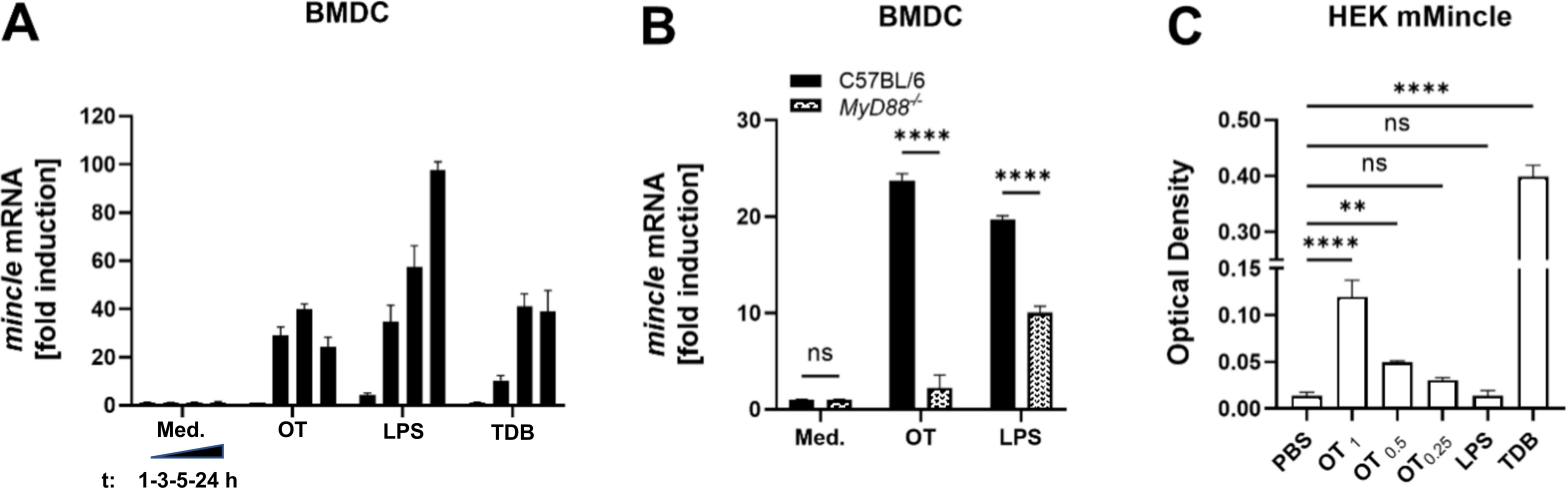
Heat-killed *Orientia* induces *mincle* mRNA via MyD88. (**A**) BMDC from C57BL/6 mice were stimulated with purified, 70°C-inactivated *Orientia*, LPS (1.0 µg/mL), TDB (10.0 µg/mL), or medium. Induction of *mincle* mRNA (normalized to RPS9) after 1, 3, 5, and 24 h was measured by RT-PCR. Data represent means ± SD of technical duplicates from one representative out of two identical independent experiments (1, 3, 5, and 24 h) and one additional experiment with different time points (5, 12, and 24 h) showing comparable results. (**B**) BMDC from *MyD88^−/−^*-deficient and C57BL/6 mice were stimulated with purified, 70°C-inactivated *Orientia*, or with LPS (1.0 µl/mL) and medium as control. Induction of *mincle* mRNA (normalized to RPS9) after 5 h was measured by RT-PCR. Means ± SD from one representative out of four independent experiments (means ± SD of technical triplicates, ****, *P* < 0.0001; ns, no significant difference by two-way analysis of variance). (**C**) mMincle-overexpressing HEK cells were stimulated with two-fold serial dilutions of purified, 70°C-inactivated *Orientia*, and LPS (1.0 µg/mL), TDB (10 µg/mL), or medium as controls. NF-κB-induced SEAP activity was measured after 9–12 h. Means ± SD from one out of four independent experiments (three replicates per experiment). ****, *P* < 0.0001; **, *P* < 0.01; ns, no significant difference (by unpaired *t*-test).

We next investigated whether stimulation with *Orientia* induces transcription of *mincle* mRNA. By qRT-PCR, a sustainable >20-fold induction of *mincle* mRNA was measured in C57BL/6 wild-type BMDC after 3, 5, and 24 h post-stimulation with *O. tsutsugamushi* (Fig. 3A). We then studied whether the induction of *mincle* mRNA by *Orientia* required TLR-mediated signals, as suggested by previous studies (45). If CLR ligation results in feed-forward signaling, the induction of *mincle* would be maintained in the absence of TLR signaling, as shown by others (46). As depicted in Fig. 3B, the induction of *mincle* mRNA by *Orientia* was abrogated in *MyD88^−/−^* BMDC, suggesting that TLR ligands from *Orientia* drive *mincle* transcription.

### Functional engagement of Mincle by *O. tsutsugamushi*

Next, we investigated whether the Mincle ligand in *Orientia* is functionally active. To that end, we used HEK cells expressing mouse Mincle together with an NF-κB-inducible reporter system (HEK-mMincle), which, upon activation, leads to the secretion of secreted alkaline phosphatase (SEAP) detectable by a chromogenic substrate. Indeed, heat-killed *O. tsutsugamushi* activated HEK-mMincle in a dose-dependent manner (Fig. 3C). In control stimulations, HEK-mMincle cells also reacted to TDB but not to LPS, demonstrating specificity of the reporter assay. Thus, heat-inactivated *Orientia* contains a component that functionally engages Mincle in a reporter assay, consistent with the presence of an agonistic ligand that is resistant to heat.

### Induction of TNF-α by *Orientia* is independent of Mincle

**Fig 4:**
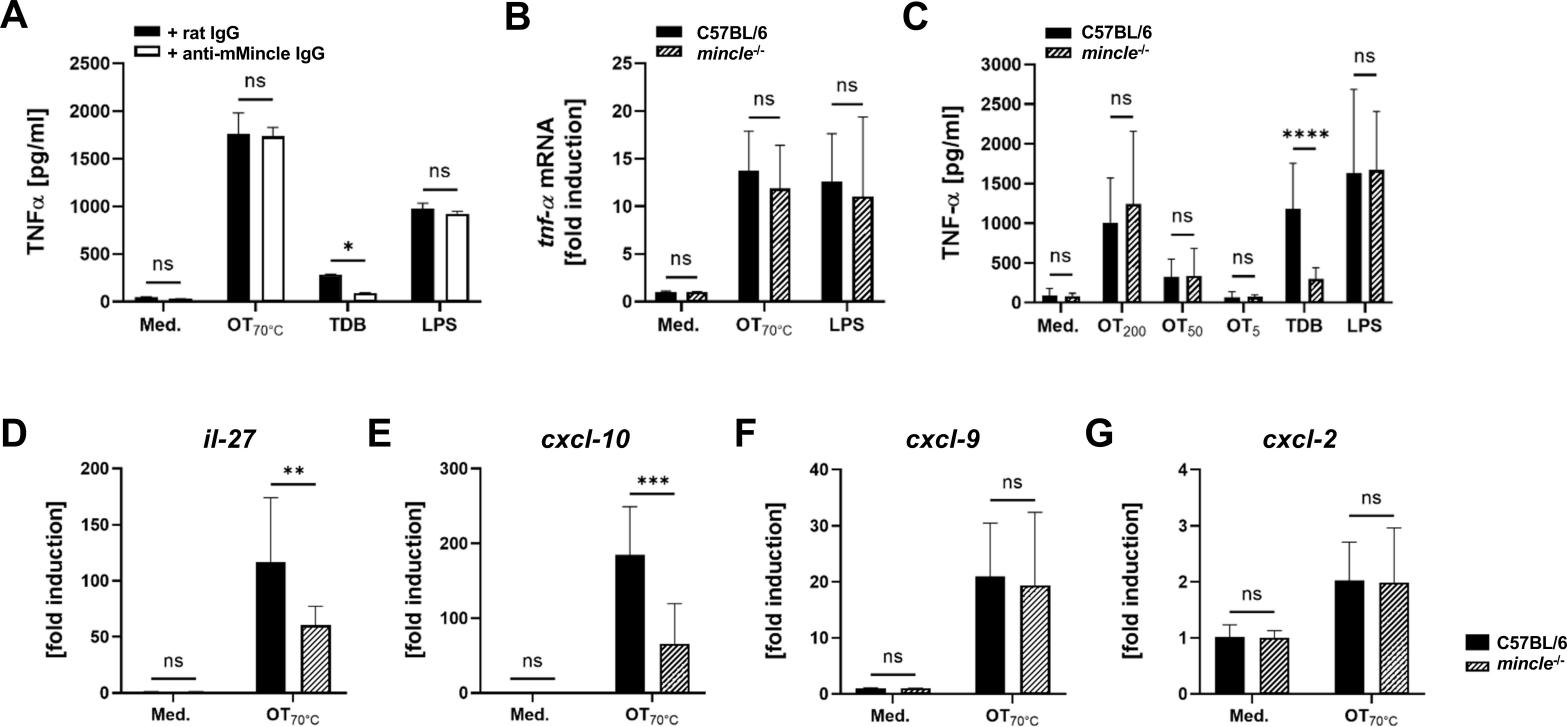
Heat-killed *Orientia* induces *il-27* and *cxcl-10* mRNA in BMDC via Mincle, but not TNF-α. (**A**) In BMDC from C57BL/6 mice, Mincle was neutralized by monoclonal Anti-mMincle IgG, or rat IgG antibody as a control (both 1.0 µg/mL), for 1 h. Cells were then stimulated with purified, 70°C-inactivated *Orientia*, TDB (10 µg/mL), LPS (1.0 µg/mL), or medium. TNF-α concentrations in supernatants were measured by ELISA 24 h post-stimulation. Means ± SD from one out of three independent experiments with three replicates per condition. (**B, C**) BMDC from C57BL/6 or *mincle^−/−^* mice were stimulated with purified, 70°C-inactivated *Orientia*, LPS (1.0 µl/mL), or medium. (**B**) Induction of *tnf-α* mRNA (normalized to RPS9) after 5 h was measured by RT-PCR. Data were pooled from three independent experiments with two replicates per condition; means ± SD. (**C**) TNF-α concentrations were measured from supernatants 24 h post-stimulation with increasing concentrations of *Orientia* (5–200/well) by ELISA. Data were pooled from five independent experiments with three replicates per condition; means ± SD. (**D–G**) BMDC from C57BL/6 and *mincle^−/−^* mice were stimulated with purified, 70°C-inactivated *Orientia* or medium. Induction of *il-27* (**D**), *cxcl-10* (**E**), *cxcl-9* (**F**), and *ccl-2* (**G**) mRNA (normalized to *rps9*) after 5 h was measured by qRT-PCR. Data obtained in three independent experiments with two replicates per condition were pooled; means ± SD. ****, *P* < 0.0001; ***, *P* < 0.001; **, *P* < 0.01; *, *P* < 0.05; ns, no significant difference by two-way analysis of variance.

Induction of TNF-α by *Orientia* serves as a hallmark of innate activation (47). We therefore investigated whether Mincle signaling contributes to induction of TNF-α in BMDC. First, we blocked Mincle by pre-treatment with a Mincle-specific IgG mAb for 1 h prior to addition of stimuli. As expected, blocking of Mincle resulted in significant reduction of TNF-α secretion by BMDC in response to the Mincle ligand TDB, while the TNF-α response to LPS was unaffected by Mincle blockade. Of note, the induction of TNF-α toward stimulation with heat-inactivated *Orientia* could not be blocked by anti-Mincle IgG (Fig. 4A). We then used BMDC derived from genetically Mincle-deficient and C57BL/6 mice and stimulated these with decreasing concentrations of heat-killed *Orientia*. As shown in Fig. 4B, there was no significant difference in the induction of *tnf-a* mRNA in response to stimulation with *Orientia*. Also, the secretion of TNF-α to supernatants did not differ significantly between BMDC from C57BL/6 and Mincle^−/−^ mice (Fig. 4C). Thus, while heat-stable ligands of *Orientia* induced Mincle via TLR pathways, these ligands did not contribute to the induction of TNF-α.

### Reduced induction of *il-27* and *cxcl-10* mRNA by *Orientia* in the absence of Mincle

We reasoned that ligation of Mincle by heat-inactivated *Orientia* may not act as a strong pro-inflammatory innate stimulus but rather regulate the transcription of certain genes. A previous study focusing on the situation in *Orientia* infection reported that the induction of IL-27 as well as the chemokines CXCL-9 and CXCL-10 were induced to a lower degree in Mincle-deficient macrophages compared to the wild type (34). In the present study, the induction of *il-27* and *cxcl-10* mRNA transcripts in response to heat-inactivated *Orientia* was reduced in Mincle^−/−^ BMDC compared to the C57BL/6 wild type (Fig. 4D and E). In contrast, transcription of *cxcl-9* and *ccl2* was unaltered in Mincle^−/−^ BMDC (Fig. 4F and G). Thus, the ability of *Orientia* to drive IL-27 and CXCL-10 responses via Mincle is retained upon heat inactivation, suggesting that microbial viability is not required for this immunomodulatory effect.

Taken together, this study demonstrates for the first time a direct binding of mouse and human CLRs to *Orientia* and particularly the Ca^2+^-dependent binding of Mincle to the pathogen. The induction of *mincle* mRNA in response to heat-inactivated *Orientia* depended on TLR signals. While we demonstrated the presence of a functional Mincle ligand in *Orientia*, this ligand did not mediate a strong induction of TNF-α in response to heat-inactivated bacteria. However, the induction of *il-27* and *cxcl-10* mRNAs was reduced in the absence of Mincle. Thus, Mincle and potentially other CLRs contribute to innate recognition of *Orientia* and shape innate inflammation in response to this obligate intracellular pathogen.

## DISCUSSION

The present study demonstrates for the first time that *Orientia* binds directly to several mouse CLRs and human DC-SIGN. To this end, a library of recombinant CLR-Fc fusion proteins, which has been instrumental in the identification of other bacteria-CLR interactions (35), was combined with an *Orientia*-/56 kD-specific monoclonal antibody (43) for flow cytometric measurements.

Prior to this report, data on the interaction of CLRs with *Orientia* had been scarce. In an earlier study, Mincle was found to be expressed in both infected and uninfected cells in mouse lungs but with limited evidence for bacteria-Mincle colocalization (34). We now show that *Orientia* binds directly to several mouse CLRs, including Mincle (Clec4e), Dectin-1 (Clec7a), and mLangerin (Clec4k). Binding to DCL-1 (Clec13a) was weaker, while more variable, non-significant binding to mouse Dectin-2 (Clec4n) and no binding to DNGR-1 (Clec9a) was found. The binding of *Orientia* to Mincle was moreover abrogated by addition of the Ca^2+^-chelating agent EDTA, demonstrating Ca^2+^ dependency of the binding (48, 49). Also, DC-SIGN was revealed as the first human CLR that strongly binds to *Orientia*.

The present study also shows that stimulation of BMDC with heat-inactivated *Orientia* is sufficient to drive the induction of *mincle* mRNA, independently of active infection. This induction requires the TLR pathway, potentially via TLR2 (7), thus adding to observations by Fisher et al. (50) that CLR-mediated signals can drive Mincle transcription.

Collaboration between TLR- and CLR-mediated signals is known to either synergistically enhance NF-κB signaling and cytokine induction (51–55), or to polarize cytokine patterns, exemplified by the reciprocal regulation of IL-12 and IL-23 after co-activation of Dectin-1 and TLR2 (55, 56). Such cross-pathway collaborations can entail the formation of CLR:TLR heterodimers, as shown for Clec2d and TLR2 (57), or the formation of partially overlapping, non-colocalized CLR:TLR receptor nanoclusters with enhanced signaling competency (58). As demonstrated here by two independent approaches, Mincle was not required for TNF-α induction after stimulation with heat-inactivated *Orientia*, confirming similar observations during *Orientia* infection in macrophages (34). In fact, the relative contribution of Mincle ligands to TNF-α responses in other infection contexts is often minor and may be overridden by TLR ligands (15). Instead, shifted patterns of cytokine responses to complex innate ligands are a typical hallmark of myeloid cells deficient for Mincle and other CLRs (15, 34, 55). We therefore investigated additional cytokines and chemokines upregulated in *Orientia* infection and found that the heat-stable ligand of *Orientia* was sufficient to induce *il-27* and *cxcl-10* mRNAs in a Mincle-dependent fashion in BMDC. IL-27 is a member of the IL-12 family of cytokines and shapes adaptive immune responses *in vivo*, by promoting early Th1 activation, augmenting effector CD8^+^ T-cell function (59, 60), and inhibiting IL-17 responses (61, 62). CXCL-10 is an IFN-γ-inducible, CXCR3-binding chemokine that also drives type 1 immunity by supporting priming of Th1 and cytotoxic T cells within draining lymph nodes and promoting efficient recruitment of effector T cells to sites of inflammation (63, 64). Type 1 immunity is a central determinant of *Orientia* infection also in experimentally infected mice and strongly linked to protective host control (65–67). Mincle-dependent induction of IL-27 and CXCL-10 could therefore contribute to sustaining Th1/CD8-dominated responses, which deserves further investigation. In sum, our data support a model of hierarchical TLR:CLR cross talk in which initial TLR activation licenses Mincle expression and function, allowing cytokine fine-tuning on the innate level.

It is interesting to note that two of the three CLRs with strong binding to *Orientia*, Mincle and Dectin-1 (summary in Table S1), are inversely regulated during infection: while Mincle is strongly upregulated, Dectin-1 is concomitantly downregulated (34, 50). Dectin-1 is a known driver of IL-17 immunity in fungal infections (68), but *Orientia* infection is characterized by IFNγ-dominated Th1 immunity and absence of IL-17 (1, 34, 43, 66, 69, 70). It remains to be investigated if the differential regulation of Mincle and Dectin-1, both binding *Orientia*, helps to maintain the type 1 inflammation milieu and avoid IL-17 responses, for example, as part of a host defense strategy or as a consequence of pathogen-driven immune modulation. Of note, the induction of Mincle was also related to *Orientia* pathogenicity, as the highly pathogenic Karp strain induced more sustained upregulation of Mincle mRNA compared to the low-pathogenic Gilliam strain (71). The higher Mincle expression induced by Karp could be, potentially via IL-27 or CXCL-10, among the factors driving the polarization of cytokine responses, such as higher IFNγ transcription observed in Karp infection.

The biochemical nature of the putative Mincle ligand in *Orientia* remains elusive. The majority of Mincle ligands from microorganisms are amphiphilic glycolipids (72) or glycoproteins (73, 74). Although several sugar moieties can bind to Mincle, there is an increased affinity to trehalose (49), and the synthesis of several polyacyl trehalose glycolipid compounds by *Orientia* has recently been predicted (75). Identification and characterization of a distinct *Orientia*-derived ligand will require purification of large amounts of candidate compounds from the cell wall of *Orientia*.

Natural engagement of CLRs by microbial glycans has informed vaccine adjuvant design in the past, as exemplified by the synthetic Mincle agonist TDB in CAF01 for eliciting balanced Th1/Th17 immunity (76); whether *Orientia*-derived CLR ligands can similarly inspire vaccine development, or whether deliberate CLR targeting will benefit vaccines against *Orientia*, remains to be investigated.

In humans, the here-identified *Orientia*-binding receptor DC-SIGN is expressed by DCs, including dermal DCs (77). In the eschar, the primary cutaneous lesion, *Orientia* infects cells that strongly express DC-SIGN (78). The role of DC-SIGN is known to depend on the nature of the microbial ligand and can entail phagocytosis and infection on one hand, or modulation of pathogen-induced cytokine patterns on the other hand (32, 33, 79, 80). Whether it contributes to either mechanism in *Orientia* infection remains to be investigated.

In conclusion, the present work is the first to demonstrate direct binding of *Orientia* to several mouse and one human CLRs; it specifically shows that TLR signals drive the expression of Mincle as a newly identified binding partner which skews the cytokine pattern toward type 1 immunity. These findings add to the innate receptor interactome of *Orientia* and will help to better understand immune modulation by this intracellular pathogen.

## SUPPLEMENTAL MATERIAL

Table S1 iai.00497-25-s0001.xlsx CLRs: binding to *Orientia* and regulation in infection.

ASM does not own the copyrights to Supplemental Material that may be linked to, or accessed through, an article. The authors have granted ASM a non-exclusive, world-wide license to publish the Supplemental Material files. Please contact the corresponding author directly for reuse.

## References

[1] Münch CCS, Upadhaya BP, Rayamajhee B, Adhikari A, Münch M, En-Nosse N, Kowalski K, Eickmann M, Bauer C, Manandhar KD, Keller C. 2023. Multiple Orientia clusters and Th1-skewed chemokine profile: a cross-sectional study in patients with scrub typhus from Nepal. Int J Infect Dis 128:78–87. doi:10.1016/j.ijid.2022.12.022 36566774

[2] Kim HL, Park HR, Kim CM, Cha YJ, Yun NR, Kim DM. 2019. Indicators of severe prognosis of scrub typhus: prognostic factors of scrub typhus severity. BMC Infect Dis 19:283. doi:10.1186/s12879-019-3903-9 30909868 PMC6434784

[3] Iwasaki H, Mizoguchi J, Takada N, Tai K, Ikegaya S, Ueda T. 2010. Correlation between the concentrations of tumor necrosis factor-α and the severity of disease in patients infected with Orientia tsutsugamushi. Int J Infect Dis 14:e328–e333. doi:10.1016/j.ijid.2009.06.002 19699129

[4] Cho NH, Seong SY, Huh MS, Han TH, Koh YS, Choi MS, Kim IS. 2000. Expression of chemokine genes in murine macrophages infected with Orientia tsutsugamushi. Infect Immun 68:594–602. doi:10.1128/IAI.68.2.594-602.2000 10639422 PMC97181

[5] Min CK, Yang JS, Kim S, Choi MS, Kim IS, Cho NH. 2008. Genome-based construction of the metabolic pathways of Orientia tsutsugamushi and comparative analysis within the Rickettsiales order. Comp Funct Genomics 2008:1–14. doi:10.1155/2008/623145 PMC2408715 18528528

[6] Cho N-H, Kim H-R, Lee J-H, Kim S-Y, Kim J, Cha S, Kim S-Y, Darby AC, Fuxelius H-H, Yin J, Kim JH, Kim J, Lee SJ, Koh Y-S, Jang W-J, Park K-H, Andersson SGE, Choi M-S, Kim I-S. 2007. The Orientia tsutsugamushi genome reveals massive proliferation of conjugative type IV secretion system and host–cell interaction genes. Proc Natl Acad Sci USA 104:7981–7986. doi:10.1073/pnas.0611553104 17483455 PMC1876558

[7] Gharaibeh M, Hagedorn M, Lilla S, Hauptmann M, Heine H, Fleischer B, Keller C. 2016. Toll-like receptor 2 recognizes Orientia tsutsugamushi and increases susceptibility to murine experimental scrub typhus. Infect Immun 84:3379–3387. doi:10.1128/IAI.00185-16 27620720 PMC5116716

[8] Nakayama K, Kurokawa K, Fukuhara M, Urakami H, Yamamoto S, Yamazaki K, Ogura Y, Ooka T, Hayashi T. 2010. Genome comparison and phylogenetic analysis of Orientia tsutsugamushi strains. DNA Res 17:281–291. doi:10.1093/dnares/dsq018 20682628 PMC2955711

[9] Amano KI, Tamura A, Ohashi N, Urakami H, Kaya S, Fukushi K. 1987. Deficiency of peptidoglycan and lipopolysaccharide components in Rickettsia tsutsugamushi. Infect Immun 55:2290–2292. doi:10.1128/iai.55.9.2290-2292.1987 3114150 PMC260693

[10] Cho KA, Jun YH, Suh JW, Kang JS, Choi HJ, Woo SY. 2010. Orientia tsutsugamushi induced endothelial cell activation via the NOD1-IL-32 pathway. Microb Pathog 49:95–104. doi:10.1016/j.micpath.2010.05.001 20470879

[11] Mayer S, Raulf MK, Lepenies B. 2017. C-type lectins: their network and roles in pathogen recognition and immunity. Histochem Cell Biol 147:223–237. doi:10.1007/s00418-016-1523-7 27999992

[12] Stegmann F, Lepenies B. 2024. Myeloid C-type lectin receptors in host-pathogen interactions and glycan-based targeting. Curr Opin Chem Biol 82:102521. doi:10.1016/j.cbpa.2024.102521 39214069

[13] Reis e Sousa C, Yamasaki S, Brown GD. 2024. Myeloid C-type lectin receptors in innate immune recognition. Immunity 57:700–717. doi:10.1016/j.immuni.2024.03.005 38599166

[14] Fischer S, Stegmann F, Gnanapragassam VS, Lepenies B. 2022. From structure to function – Ligand recognition by myeloid C-type lectin receptors. Comput Struct Biotechnol J 20:5790–5812. doi:10.1016/j.csbj.2022.10.019 36382179 PMC9630629

[15] Ishikawa E, Ishikawa T, Morita YS, Toyonaga K, Yamada H, Takeuchi O, Kinoshita T, Akira S, Yoshikai Y, Yamasaki S. 2009. Direct recognition of the mycobacterial glycolipid, trehalose dimycolate, by C-type lectin Mincle. J Exp Med 206:2879–2888. doi:10.1084/jem.20091750 20008526 PMC2806462

[16] Raulf MK, Johannssen T, Matthiesen S, Neumann K, Hachenberg S, Mayer-Lambertz S, Steinbeis F, Hegermann J, Seeberger PH, Baumgärtner W, Strube C, Ruland J, Lepenies B. 2019. The C-type lectin receptor CLEC12A recognizes plasmodial hemozoin and contributes to cerebral malaria development. Cell Rep 28:30–38. doi:10.1016/j.celrep.2019.06.015 31269448 PMC6616648

[17] Neumann K, Castiñeiras-Vilariño M, Höckendorf U, Hannesschläger N, Lemeer S, Kupka D, Meyermann S, Lech M, Anders HJ, Kuster B, Busch DH, Gewies A, Naumann R, Groß O, Ruland J. 2014. Clec12a is an inhibitory receptor for uric acid crystals that regulates inflammation in response to cell death. Immunity 40:389–399. doi:10.1016/j.immuni.2013.12.015 24631154

[18] Torigoe S, Schutt CR, Yamasaki S. 2021. Immune discrimination of the environmental spectrum through C-type lectin receptors. Int Immunol 33:847–851. doi:10.1093/intimm/dxab074 34599808

[19] Schoenen H, Bodendorfer B, Hitchens K, Manzanero S, Werninghaus K, Nimmerjahn F, Agger EM, Stenger S, Andersen P, Ruland J, Brown GD, Wells C, Lang R. 2010. Cutting edge: Mincle is essential for recognition and adjuvanticity of the mycobacterial cord factor and its synthetic analog trehalose-dibehenate. J Immunol 184:2756–2760. doi:10.4049/jimmunol.0904013 20164423 PMC3442336

[20] Imai T, Matsumura T, Mayer-Lambertz S, Wells CA, Ishikawa E, Butcher SK, Barnett TC, Walker MJ, Imamura A, Ishida H, Ikebe T, Miyamoto T, Ato M, Ohga S, Lepenies B, van Sorge NM, Yamasaki S. 2018. Lipoteichoic acid anchor triggers Mincle to drive protective immunity against invasive group A Streptococcus infection. Proc Natl Acad Sci USA 115:E10662–E10671. doi:10.1073/pnas.1809100115 30352847 PMC6233082

[21] Stegmann F, Diersing C, Lepenies B. 2024. Legionella pneumophila modulates macrophage functions through epigenetic reprogramming via the C-type lectin receptor Mincle. iScience 27:110700. doi:10.1016/j.isci.2024.110700 39252966 PMC11382120

[22] Kottom TJ, Hebrink DM, Carmona EM, Limper AH. 2020. Pneumocystis carinii major surface glycoprotein dampens macrophage inflammatory responses to fungal β-glucan. J Infect Dis 222:1213–1221. doi:10.1093/infdis/jiaa218 32363390 PMC7459132

[23] Yamasaki S, Matsumoto M, Takeuchi O, Matsuzawa T, Ishikawa E, Sakuma M, Tateno H, Uno J, Hirabayashi J, Mikami Y, Takeda K, Akira S, Saito T. 2009. C-type lectin Mincle is an activating receptor for pathogenic fungus, Malassezia. Proc Natl Acad Sci USA 106:1897–1902. doi:10.1073/pnas.0805177106 19171887 PMC2644135

[24] Iborra S, Martínez-López M, Cueto FJ, Conde-Garrosa R, Del Fresno C, Izquierdo HM, Abram CL, Mori D, Campos-Martín Y, Reguera RM, Kemp B, Yamasaki S, Robinson MJ, Soto M, Lowell CA, Sancho D. 2016. Leishmania uses Mincle to target an inhibitory ITAM signaling pathway in dendritic cells that dampens adaptive immunity to infection. Immunity 45:788–801. doi:10.1016/j.immuni.2016.09.012 27742545 PMC5074365

[25] Patin EC, Orr SJ, Schaible UE. 2017. Macrophage inducible C-type lectin as a multifunctional player in immunity. Front Immunol 8:1–10. doi:10.3389/fimmu.2017.00861 28791019 PMC5525440

[26] Saijo S, Fujikado N, Furuta T, Chung S, Kotaki H, Seki K, Sudo K, Akira S, Adachi Y, Ohno N, Kinjo T, Nakamura K, Kawakami K, Iwakura Y. 2007. Dectin-1 is required for host defense against Pneumocystis carinii but not against Candida albicans. Nat Immunol 8:39–46. doi:10.1038/ni1425 17159982

[27] Yonekawa A, Saijo S, Hoshino Y, Miyake Y, Ishikawa E, Suzukawa M, Inoue H, Tanaka M, Yoneyama M, Oh-Hora M, Akashi K, Yamasaki S. 2014. Dectin-2 is a direct receptor for mannose-capped lipoarabinomannan of mycobacteria. Immunity 41:402–413. doi:10.1016/j.immuni.2014.08.005 25176311

[28] Rothfuchs AG, Bafica A, Feng CG, Egen JG, Williams DL, Brown GD, Sher A. 2007. Dectin-1 interaction with Mycobacterium tuberculosis leads to enhanced IL-12p40 production by splenic dendritic cells. J Immunol 179:3463–3471. doi:10.4049/jimmunol.179.6.3463 17785780

[29] Taylor PR, Tsoni SV, Willment JA, Dennehy KM, Rosas M, Findon H, Haynes K, Steele C, Botto M, Gordon S, Brown GD. 2007. Dectin-1 is required for β-glucan recognition and control of fungal infection. Nat Immunol 8:31–38. doi:10.1038/ni1408 17159984 PMC1888731

[30] de Witte L, Nabatov A, Pion M, Fluitsma D, de Jong MAWP, de Gruijl T, Piguet V, van Kooyk Y, Geijtenbeek TBH. 2007. Langerin is a natural barrier to HIV-1 transmission by Langerhans cells. Nat Med 13:367–371. doi:10.1038/nm1541 17334373

[31] Hunger RE, Sieling PA, Ochoa MT, Sugaya M, Burdick AE, Rea TH, Brennan PJ, Belisle JT, Blauvelt A, Porcelli SA, Modlin RL. 2004. Langerhans cells utilize CD1a and langerin to efficiently present nonpeptide antigens to T cells. J Clin Invest 113:701–708. doi:10.1172/JCI19655 14991068 PMC351318

[32] Gringhuis SI, den Dunnen J, Litjens M, van het Hof B, van Kooyk Y, Geijtenbeek TBH. 2007. C-type lectin DC-SIGN modulates toll-like receptor signaling via Raf-1 kinase-dependent acetylation of transcription factor NF-κB. Immunity 26:605–616. doi:10.1016/j.immuni.2007.03.012 17462920

[33] Geijtenbeek TBH, Van Vliet SJ, Koppel EA, Sanchez-Hernandez M, Vandenbroucke-Grauls CMJE, Appelmelk B, Van Kooyk Y. 2003. Mycobacteria target DC-SIGN to suppress dendritic cell function. J Exp Med 197:7–17. doi:10.1084/jem.20021229 12515809 PMC2193797

[34] Fisher J, Card G, Liang Y, Trent B, Rosenzweig H, Soong L. 2021. Orientia tsutsugamushi selectively stimulates the C-type lectin receptor Mincle and type 1-skewed proinflammatory immune responses. PLoS Pathog 17:e1009782. doi:10.1371/journal.ppat.1009782 34320039 PMC8351992

[35] Mayer S, Moeller R, Monteiro JT, Ellrott K, Josenhans C, Lepenies B. 2018. C-type lectin receptor (CLR)-Fc fusion proteins as tools to screen for novel CLR/bacteria interactions: an exemplary study on preselected Campylobacter jejuni isolates. Front Immunol 9:213. doi:10.3389/fimmu.2018.00213 29487596 PMC5816833

[36] Maglinao M, Eriksson M, Schlegel MK, Zimmermann S, Johannssen T, Götze S, Seeberger PH, Lepenies B. 2014. A platform to screen for C-type lectin receptor-binding carbohydrates and their potential for cell-specific targeting and immune modulation. J Control Release 175:36–42. doi:10.1016/j.jconrel.2013.12.011 24368301

[37] Helk E, Bernin H, Ernst T, Ittrich H, Jacobs T, Heeren J, Tacke F, Tannich E, Lotter H. 2013. TNFα-mediated liver destruction by Kupffer cells and Ly6Chi monocytes during Entamoeba histolytica infection. PLoS Pathog 9:e1003096. doi:10.1371/journal.ppat.1003096 23300453 PMC3536671

[38] Hu C, Ding H, Li Y, Pearson JA, Zhang X, Flavell RA, Wong FS, Wen L. 2015. NLRP3 deficiency protects from type 1 diabetes through the regulation of chemotaxis into the pancreatic islets. Proc Natl Acad Sci USA 112:11318–11323. doi:10.1073/pnas.1513509112 26305961 PMC4568693

[39] Valbuena G, Bradford W, Walker DH. 2003. Expression analysis of the T-cell-targeting chemokines CXCL9 and CXCL10 in mice and humans with endothelial infections caused by rickettsiae of the spotted fever group. Am J Pathol 163:1357–1369. doi:10.1016/S0002-9440(10)63494-3 14507644 PMC1868304

[40] Harker JA, Wong KA, Dallari S, Bao P, Dolgoter A, Jo Y, Wehrens EJ, Macal M, Zuniga EI, Harker CJ. 2018. Interleukin-27R signaling mediates early viral containment and impacts innate and adaptive immunity after chronic lymphocytic choriomeningitis virus infection. J Virol 92:e02196–02217. doi:10.1128/JVI.02196-17 29593047 PMC5974502

[41] Wells CA, Salvage-Jones JA, Li X, Hitchens K, Butcher S, Murray RZ, Beckhouse AG, Lo Y-L-S, Manzanero S, Cobbold C, Schroder K, Ma B, Orr S, Stewart L, Lebus D, Sobieszczuk P, Hume DA, Stow J, Blanchard H, Ashman RB. 2008. The macrophage-inducible C-type lectin, mincle, is an essential component of the innate immune response to Candida albicans. J Immunol 180:7404–7413. doi:10.4049/jimmunol.180.11.7404 18490740

[42] Petermann M, Orfanos Z, Sellau J, Gharaibeh M, Lotter H, Fleischer B, Keller C. 2021. CCR2 deficiency impairs Ly6Clo and Ly6Chi monocyte responses in Orientia tsutsugamushi infection. Front Immunol 12:1–15. doi:10.3389/fimmu.2021.670219 PMC8287586 34290699

[43] Keller CA, Hauptmann M, Kolbaum J, Gharaibeh M, Neumann M, Glatzel M, Fleischer B. 2014. Dissemination of Orientia tsutsugamushi and inflammatory responses in a murine model of scrub typhus. PLoS Negl Trop Dis 8:e3064. doi:10.1371/journal.pntd.0003064 25122501 PMC4133189

[44] Kim MK, Kang JS. 2001. Orientia tsutsugamushi suppresses the production of inflammatory cytokines induced by its own heat-stable component in murine macrophages. Microb Pathog 31:145–150. doi:10.1006/mpat.2001.0457 11500099

[45] Kerscher B, Wilson GJ, Reid DM, Mori D, Taylor JA, Besra GS, Yamasaki S, Willment JA, Brown GD. 2016. Mycobacterial receptor, Clec4d (CLECSF8, MCL), is coregulated with Mincle and upregulated on mouse myeloid cells following microbial challenge. Eur J Immunol 46:381–389. doi:10.1002/eji.201545858 26558717 PMC4833188

[46] Schoenen H, Huber A, Sonda N, Zimmermann S, Jantsch J, Lepenies B, Bronte V, Lang R. 2014. Differential control of Mincle-dependent cord factor recognition and macrophage responses by the transcription factors C/EBPβ and HIF1α. J Immunol 193:3664–3675. doi:10.4049/jimmunol.1301593 25156364

[47] Kim MJ, Kim MK, Kang JS. 2006. Orientia tsutsugamushi inhibits tumor necrosis factor alpha production by inducing interleukin 10 secretion in murine macrophages. Microb Pathog 40:1–7. doi:10.1016/j.micpath.2005.09.002 16325368

[48] Matsumoto M, Tanaka T, Kaisho T, Sanjo H, Copeland NG, Gilbert DJ, Jenkins NA, Akira S. 1999. A novel LPS-inducible C-type lectin is a transcriptional target of NF-IL6 in macrophages. J Immunol 163:5039–5048. doi:10.4049/jimmunol.163.9.5039 10528209

[49] Feinberg H, Rambaruth NDS, Jégouzo SAF, Jacobsen KM, Djurhuus R, Poulsen TB, Weis WI, Taylor ME, Drickamer K. 2016. Binding sites for acylated trehalose analogs of glycolipid ligands on an extended carbohydrate recognition domain of the macrophage receptor Mincle. J Biol Chem 291:21222–21233. doi:10.1074/jbc.M116.749515 27542410 PMC5076529

[50] Fisher J, Gonzales C, Chroust Z, Liang Y, Soong L. 2023. Orientia tsutsugamushi infection stimulates Syk-dependent responses and innate cytosolic defenses in macrophages. Pathogens 12:53. doi:10.3390/pathogens12010053 PMC9861896 36678402

[51] Matsumura T, Ikebe T, Arikawa K, Hosokawa M, Aiko M, Iguchi A, Togashi I, Kai S, Ohara S, Ohara N, Ohnishi M, Watanabe H, Kobayashi K, Takeyama H, Yamasaki S, Takahashi Y, Ato M. 2019. Sequential sensing by TLR2 and Mincle directs immature myeloid cells to protect against invasive group A streptococcal infection in mice. Cell Rep 27:561–571. doi:10.1016/j.celrep.2019.03.056 30970258

[52] Schick J, Etschel P, Bailo R, Ott L, Bhatt A, Lepenies B, Kirschning C, Burkovski A, Lang R. 2017. Toll-like receptor 2 and Mincle cooperatively sense corynebacterial cell wall glycolipids. Infect Immun 85:e00075-17. doi:10.1128/IAI.00075-17 28483856 PMC5478951

[53] Sakabe R, Onishi K, Mochizuki J, Toshimitsu T, Shimazu T, Kishino S, Ogawa J, Yamasaki S, Sashihara T. 2025. Regulation of IL-10 production in dendritic cells is controlled by the co-activation of TLR2 and Mincle by Lactiplantibacillus plantarum OLL2712. Microbiol Spectr 13:e0119624. doi:10.1128/spectrum.01196-24 39902909 PMC11878067

[54] Gantner BN, Simmons RM, Canavera SJ, Akira S, Underhill DM. 2003. Collaborative induction of inflammatory responses by dectin-1 and Toll-like receptor 2. J Exp Med 197:1107–1117. doi:10.1084/jem.20021787 12719479 PMC2193968

[55] Dennehy KM, Willment JA, Williams DL, Brown GD. 2009. Reciprocal regulation of IL-23 and IL-12 following co-activation of Dectin-1 and TLR signaling pathways. Eur J Immunol 39:1379–1386. doi:10.1002/eji.200838543 19291703 PMC2720084

[56] Underhill DM. 2007. Collaboration between the innate immune receptors dectin-1, TLRs, and Nods. Immunol Rev 219:75–87. doi:10.1111/j.1600-065X.2007.00548.x 17850483

[57] Li F, Wang H, Li YQ, Gu Y, Jia XM. 2023. C-type lectin receptor 2d forms homodimers and heterodimers with TLR2 to negatively regulate IRF5-mediated antifungal immunity. Nat Commun 14. doi:10.1038/s41467-023-42216-3 PMC10593818 37872182

[58] Li M, Vultorius C, Bethi M, Yu Y. 2022. Spatial organization of dectin-1 and TLR2 during synergistic crosstalk revealed by super-resolution imaging. J Phys Chem B 126:5781–5792. doi:10.1021/acs.jpcb.2c03557 35913832 PMC10636754

[59] Morishima N, Owaki T, Asakawa M, Kamiya S, Mizuguchi J, Yoshimoto T. 2005. Augmentation of effector CD8+ T cell generation with enhanced granzyme B expression by IL-27. J Immunol 175:1686–1693. doi:10.4049/jimmunol.175.3.1686 16034109

[60] Pflanz S, Timans JC, Cheung J, Rosales R, Kanzler H, Gilbert J, Hibbert L, Churakova T, Travis M, Vaisberg E, Blumenschein WM, Mattson JD, Wagner JL, To W, Zurawski S, McClanahan TK, Gorman DM, Bazan JF, de Waal Malefyt R, Rennick D, Kastelein RA. 2002. IL-27, a heterodimeric cytokine composed of EBI3 and p28 protein, induces proliferation of naive CD4+ T cells. Immunity 16:779–790. doi:10.1016/s1074-7613(02)00324-2 12121660

[61] Diveu C, McGeachy MJ, Boniface K, Stumhofer JS, Sathe M, Joyce-Shaikh B, Chen Y, Tato CM, McClanahan TK, de Waal Malefyt R, Hunter CA, Cua DJ, Kastelein RA. 2009. IL-27 blocks RORc expression to inhibit lineage commitment of Th17 cells. J Immunol 182:5748–5756. doi:10.4049/jimmunol.0801162 19380822

[62] Amadi-Obi A, Yu CR, Liu X, Mahdi RM, Clarke GL, Nussenblatt RB, Gery I, Lee YS, Egwuagu CE. 2007. TH17 cells contribute to uveitis and scleritis and are expanded by IL-2 and inhibited by IL-27/STAT1. Nat Med 13:711–718. doi:10.1038/nm1585 17496900

[63] Yoneyama H, Narumi S, Zhang Y, Murai M, Baggiolini M, Lanzavecchia A, Ichida T, Asakura H, Matsushima K. 2002. Pivotal role of dendritic cell-derived CXCL10 in the retention of T helper cell 1 lymphocytes in secondary lymph nodes. J Exp Med 195:1257–1266. doi:10.1084/jem.20011983 12021306 PMC2193754

[64] Dufour JH, Dziejman M, Liu MT, Leung JH, Lane TE, Luster AD. 2002. IFN-γ-inducible protein 10 (IP-10; CXCL10)-deficient mice reveal a role for IP-10 in effector T cell generation and trafficking. J Immunol 168:3195–3204. doi:10.4049/jimmunol.168.7.3195 11907072

[65] Xu G, Mendell NL, Liang Y, Shelite TR, Goez-Rivillas Y, Soong L, Bouyer DH, Walker DH. 2017. CD8+ T cells provide immune protection against murine disseminated endotheliotropic Orientia tsutsugamushi infection. PLoS Negl Trop Dis 11:e0005763. doi:10.1371/journal.pntd.0005763 28723951 PMC5536391

[66] Hauptmann M, Kolbaum J, Lilla S, Wozniak D, Gharaibeh M, Fleischer B, Keller CA. 2016. Protective and pathogenic roles of CD8+ T lymphocytes in murine Orientia tsutsugamushi infection. PLoS Negl Trop Dis 10:e0004991. doi:10.1371/journal.pntd.0004991 27606708 PMC5015871

[67] Soong L, Wang H, Shelite TR, Liang Y, Mendell NL, Sun J, Gong B, Valbuena GA, Bouyer DH, Walker DH. 2014. Strong type 1, but impaired type 2, immune responses contribute to Orientia tsutsugamushi-induced pathology in mice. PLoS Negl Trop Dis 8:e3191. doi:10.1371/journal.pntd.0003191 25254971 PMC4177881

[68] LeibundGut-Landmann S, Groß O, Robinson MJ, Osorio F, Slack EC, Tsoni SV, Schweighoffer E, Tybulewicz V, Brown GD, Ruland J, Reis e Sousa C. 2007. Syk- and CARD9-dependent coupling of innate immunity to the induction of T helper cells that produce interleukin 17. Nat Immunol 8:630–638. doi:10.1038/ni1460 17450144

[69] Soong L, Shelite HTR, Xing Y, Kodakandla H, Liang Y, Trent BJ, Horton P, Smith KC, Zhao Z, Sun J, Bouyer DH, Cai J. 2017. Type 1-skewed neuroinflammation and vascular damage associated with Orientia tsutsugamushi infection in mice. PLoS Negl Trop Dis 11:e0005765. doi:10.1371/journal.pntd.0005765 28742087 PMC5542690

[70] Kang SJ, Jin HM, Cho YN, Kim SE, Kim UJ, Park KH, Jang HC, Jung SI, Kee SJ, Park YW. 2017. Increased level and interferon-γ production of circulating natural killer cells in patients with scrub typhus. PLoS Negl Trop Dis 11:1–19. doi:10.1371/journal.pntd.0005815 PMC5549767 28750012

[71] Thiriot JD, Liang Y, Gonzales C, Sun J, Yu X, Soong L. 2023. Differential cellular immune responses against Orientia tsutsugamushi Karp and Gilliam strains following acute infection in mice. PLoS Negl Trop Dis 17:e0011445. doi:10.1371/journal.pntd.0011445 38091346 PMC10752558

[72] Lu X, Nagata M, Yamasaki S. 2018. Mincle: 20 years of a versatile sensor of insults. Int Immunol 30:233–239. doi:10.1093/intimm/dxy028 29726997

[73] Chinthamani S, Settem RP, Honma K, Kay JG, Sharma A. 2017. Macrophage inducible C-type lectin (Mincle) recognizes glycosylated surface (S)-layer of the periodontal pathogen Tannerella forsythia. PLoS One 12:e0173394. doi:10.1371/journal.pone.0173394 28264048 PMC5338828

[74] Malamud M, Carasi P, Assandri MH, Freire T, Lepenies B, Serradell M de L. 2019. S-layer glycoprotein from Lactobacillus kefiri exerts its immunostimulatory activity through glycan recognition by Mincle. Front Immunol 10:1–11. doi:10.3389/fimmu.2019.01422 31297112 PMC6607945

[75] Karp PD, Billington R, Caspi R, Fulcher CA, Latendresse M, Kothari A, Keseler IM, Krummenacker M, Midford PE, Ong Q, Ong WK, Paley SM, Subhraveti P. 2019. The BioCyc collection of microbial genomes and metabolic pathways. Brief Bioinformatics 20:1085–1093. doi:10.1093/bib/bbx085 29447345 PMC6781571

[76] Weth AF, Dangerfield EM, Timmer MSM, Stocker BL. 2024. Recent advances in the development of Mincle-targeting vaccine adjuvants. Vaccines (Basel) 12:1320. doi:10.3390/vaccines12121320 39771982 PMC11680293

[77] Geijtenbeek TBH, Torensma R, van Vliet SJ, van Duijnhoven GCF, Adema GJ, van Kooyk Y, Figdor CG. 2000. Identification of DC-SIGN, a novel dendritic cell-specific ICAM-3 receptor that supports primary immune responses. Cell 100:575–585. doi:10.1016/S0092-8674(00)80693-5 10721994

[78] Paris DH, Phetsouvanh R, Tanganuchitcharnchai A, Jones M, Jenjaroen K, Vongsouvath M, Ferguson DPJ, Blacksell SD, Newton PN, Day NPJ, Turner GDH. 2012. Orientia tsutsugamushi in human scrub typhus eschars shows tropism for dendritic cells and monocytes rather than endothelium. PLoS Negl Trop Dis 6:e1466. doi:10.1371/journal.pntd.0001466 22253938 PMC3254662

[79] Gringhuis SI, den Dunnen J, Litjens M, van der Vlist M, Geijtenbeek TBH. 2009. Carbohydrate-specific signaling through the DC-SIGN signalosome tailors immunity to Mycobacterium tuberculosis, HIV-1 and Helicobacter pylori. Nat Immunol 10:1081–1088. doi:10.1038/ni.1778 19718030

[80] Engering A, Geijtenbeek TBH, van Vliet SJ, Wijers M, van Liempt E, Demaurex N, Lanzavecchia A, Fransen J, Figdor CG, Piguet V, van Kooyk Y. 2002. The dendritic cell-specific adhesion receptor DC-SIGN internalizes antigen for presentation to T cells. J Immunol 168:2118–2126. doi:10.4049/jimmunol.168.5.2118 11859097

